# Progesterone receptor isoform A may regulate the effects of neoadjuvant aglepristone in canine mammary carcinoma

**DOI:** 10.1186/s12917-014-0296-2

**Published:** 2014-12-17

**Authors:** Silvia Guil-Luna, Jan Stenvang, Nils Brünner, Francisco Javier De Andrés, Eva Rollón, Víctor Domingo, Raquel Sánchez-Céspedes, Yolanda Millán, Juana Martín de las Mulas

**Affiliations:** Department of Comparative Pathology, Veterinary Faculty, University of Córdoba, Edificio de Sanidad Animal. Campus de Rabanales. Carretera de Madrid-Cádiz Km. 396, 14014 Córdoba, Spain; Institute of Veterinary Disease Biology, Faculty of Health and Medical Sciences, University of Copenhagen, Copenhagen, Denmark; Small Animal Clinic Thor, Córdoba, Spain; Small Animal Clinic Canymar, Cádiz, Spain; Small Animal Clinic Recuerda, Granada, Spain

**Keywords:** Canine mammary carcinoma, Progesterone receptor, Isoforms, Aglepristone, Hormone treatment

## Abstract

**Background:**

Progesterone receptors play a key role in the development of canine mammary tumours, and recent research has focussed on their possible value as therapeutic targets using antiprogestins. Cloning and sequencing of the progesterone receptor gene has shown that the receptor has two isoforms, A and B, transcribed from a single gene. Experimental studies in human breast cancer suggest that the differential expression of progesterone receptor isoforms has implications for hormone therapy responsiveness. This study examined the effects of the antiprogestin aglepristone on cell proliferation and mRNA expression of progesterone receptor isoforms A and B in mammary carcinomas in dogs treated with 20 mg/Kg of aglepristone (n = 22) or vehicle (n = 5) twice before surgery.

**Results:**

Formalin-fixed, paraffin-embedded tissue samples taken before and after treatment were used to analyse total progesterone receptor and both isoforms by RT-qPCR and Ki67 antigen labelling. Both total progesterone receptor and isoform A mRNA expression levels decreased after treatment with aglepristone. Furthermore, a significant decrease in the proliferation index (percentage of Ki67-labelled cells) was observed in progesterone-receptor positive and isoform-A positive tumours in aglepristone-treated dogs.

**Conclusions:**

These findings suggest that the antiproliferative effects of aglepristone in canine mammary carcinomas are mediated by progesterone receptor isoform A.

**Electronic supplementary material:**

The online version of this article (doi:10.1186/s12917-014-0296-2) contains supplementary material, which is available to authorized users.

## Background

Epidemiological and clinical data indicate that progesterone has proliferative effects on normal and neoplastic canine mammary epithelium [1]. Immunohistochemical (IHC) labelling at diagnosis has identified approximately two thirds of canine mammary carcinomas as progesterone receptor (PR) positive [2]. Moreover, neoadjuvant treatment with the antiprogestin aglepristone has been found to decrease cell proliferation in PR positive canine mammary carcinomas [3]. Aglepristone is currently used in clinical practice to induce abortion and treat pyometra, as well in the treatment of proliferative progesterone-dependent diseases such as mammary fibroadenomatous hyperplasia in queens and vaginal tumours in bitches.

Like its human counterpart, canine PR exists as two isoforms: PR isoform A (PRA) and PR isoform B (PRB), which are transcribed from a single gene under the control of different promoters [4]. Under physiological conditions, normal human breast tissue expresses both PRA and PRB at equimolar levels [5]. However, an altered PRA/PRB ratio is often associated with breast carcinogenesis, PRA predominating over PRB in benign and malignant human breast tumours [5]. Findings in dogs remain controversial, due to the paucity of research and the limited number of samples analysed. Western blot analysis of normal and tumoural mammary glands from six female dogs (two in metoestrus, two in anoestrus and two after prolonged treatment with progestins) showed that PRA was either equimolar or predominant in most samples, whereas predominance of PRB was recorded in only one case [4]. Moreover, the same technique has revealed predominant staining for PRA with less intense staining for PRB in two normal canine mammary glands, three hyperplasias and three mammary carcinomas [6].

Despite their structural similarities, human PRA and PRB have been shown to have different functions, in that they regulate different subsets of genes [7]. In human breast cancer, carcinomas with higher levels of PRA than PRB were inhibited by antiprogestins, whereas carcinomas with high levels of PRB displayed no response to endocrine treatment [7]. Accordingly, it has been suggested that the relative proportion of PR isoforms A and B might affect the prognosis and thus influence therapeutic decisions [5]. We have previously shown that 1) neoadjuvant treatment with aglepristone decreases cell proliferation in PR positive carcinomas [3], and 2) PRA and PRB mRNA expression can be analysed in formalin-fixed, paraffin-embedded canine mammary gland tissue samples by RT-qPCR [8]. This study sought to examine the link between the effects of aglepristone on the proliferation index and mRNA expression of PRA and PRB in canine mammary carcinomas. IHC data of PR expression in the cases under study have been previously published [3].

## Methods

### Tissue samples and clinical data

Formalin-fixed paraffin-embedded (FFPE) tissue samples from 27 canine mammary carcinomas were taken from 27 dogs randomly recruited between 2008 and 2010 for a pharmacodynamic study [3]. Dogs were aged 5 to 16, of both pure (n = 14) and mixed (n = 13) breeds, and at all phases of the oestrous cycle except oestrus (23 anoestrus, 3 dioestrus and 1 proestrus) as determined by vaginal cytology. Carcinomas were at three clinical stages: I (n = 19), II (n = 6) and III (n = 2). None of the dogs had lung metastases (as determined by two thoracic radiographs).

### Treatment protocol

All owners gave their informed consent for inclusion of their pets in this study, which did not require approval by the Bioethics Committee of the University of Córdoba (RD53/2013). All dogs received 2 subcutaneous injections of 20 mg/kg of aglepristone (Alizine, Virbac, France) (n = 22 dogs) or vehicle (n = 5 dogs) on days 1 and 7, Surgery was performed on day 15 [3]. To analyse the effects of aglepristone, a core biopsy was taken on day 1 prior to the first injection of aglepristone or oil vehicle, and the biopsied area was marked with suture thread in order to avoid variability in subsequent studies. Finally, all patients underwent complete surgical excision of the tumour on day 15 [3]. Fixation time ranged from 24 to 48 hours, and paraffin blocks were stored at 4°C. Histological tumour types [9] included 14 complex carcinomas, 6 simple carcinomas, 5 carcinomas in benign tumour, 1 carcinosarcoma and 1 squamous cell carcinoma.

### RT-qPCR analysis

PR expression was analysed before and after treatment (treated and control samples). RNA was isolated using the RNase FFPE kit (Qiagen, Copenhagen, Denmark) in accordance with manufacturers’ recommendations. RNA yields and purity were determined by spectrophotometric absorbance at 260 nm (A_260_) measured with a NanoDrop® ND-1000 spectrophotometer (Thermo Scientific, Wilmington, DE, USA). A ratio of absorbance at 260 nm and 280 nm 1.8-2.0 was accepted as “pure”. The integrity of total RNA was checked by denaturing agarose gel electrophoresis and ethidium bromide staining showing the respective mRNAs as sharp bands.

Extracted RNA was stored at −80°C until use. It was then amplified and melting-curve analysis was carried out using the LightCycler® 480 Real-Time PCR System. One-step RT-qPCR was performed using the QuantiFast® SYBR® Green RT-PCR (Qiagen, Copenhagen, Denmark), following a previously-described protocol [8]. Canine-specific primers for PR gene were designed specifically to target the region of canine isoform B and the region common to both isoforms (total PR) using Primer3Plus and based on the reported canine PR sequence (NM_001003074) [4]. Primers flanking one intron were chosen wherever possible and their specificity was checked by performing a BLAST® search showing 100% homology to target genes [8]. Moreover, primers were designed to produce an amplicon smaller than 100 bp in order to ensure that the sequences were unique for the template (Table 1, Additional file 1). PR expression was normalised against two canine housekeeping genes: hypoxanthine phosphoribosyl-transferase 1 (HPTR1, NM_001003357.1) and canine ribosomal protein L32 (RPL32, NM_001252169.1). Forward and reverse primer sequences for PR gene and housekeeping genes are summarised in Table 1. Reverse transcription negative controls and non-template controls were included, and PCR products were separated in a 3% agarose gel and visualised by ethidium bromide staining.

**Table 1:** **Primer sequences for RT-qPCR amplification**

	**Primer Forward**	**Primer Reverse**	**Product length**
**PR**	5′-GGCTTGCCGCAGGTGTACCA-3′	5′- ACTGTGGGCTCTGGCTGGCA-3′	73 bp
**PRB**	5′-CCCGGGCGGATCCGAGACT-3′	5′-GTGCAGCGGCCCTCGGTC-3′	86 bp
**HPTR1**	5′-TGCAGACTTTGCTTTCCTTGGTCA-3′	5′-TCGAGGGGTCCTTTTCACCAGCA -3′	81 bp
**RPL32**	5′-GGCTGCCCTCAGACCTCTGGT -3′	5′-TCGGTCTGACTGGTGCCGGA -3′	79 bp

PR: Progesterone receptor; PRB: Progesterone receptor isoform B; HPTR1:Hypoxanthine phosphoribosyl-transferase 1; RPL32: Canine ribosomal protein L32.

For relative quantitation, target gene signals were normalised against those of the two selected housekeeping genes using the comparative Ct method (∆∆Ct) following Schmittgen et al. [10]. RT-qPCR data was presented as 2^-∆Ct^ where ΔCt = Ct_TARGET_ –Ct_RPL32/HPTR1,_ and Ct_RPL32/HPTR1_ is the geometric mean of the Ct values of the two housekeeping genes for each sample. PRA levels were calculated by subtracting the relative amount of PRB from that of total PR, as reported in studies in humans [11].

Amplification plots derived from melting-curve analyses displayed satisfactory amplification curves, single-peak melting curves and adequate melting temperatures. For PRB, however, melting-curve analysis revealed one major sharp peak but also additional extra minor peaks at lower Tm consistent with the agarose gel [8]. Concordance between data of immunohistochemistry and gene expression data was calculated using Cohen’s Kappa statistics.

### Proliferation index (PI)

The proliferation index was analysed by IHC in all samples obtained before and after treatment. The monoclonal mouse anti-human Ki67 antigen (clone MIB-1) isotype IgG_1_ (Dako, Barcelona, Spain) diluted 1:75, and the ABC method were applied as described previously [3]. Briefly, the slides were incubated in a water bath at 95-99°C with 0.01 M citrate buffer for 40 minutes at pH 6.0 for antigen retrieval. After cooling, sections were covered with 10% normal goat serum in PBS for 30 minutes before incubation with the primary antibody for 18 hours. The avidin-biotin-peroxidase complex was applied for 1 hour at room temperature. The chromogen, 3,3-diaminobenzidine tetra-hydrochloride (Sigma, Saint Louis, USA) diluted 0.035% in 0.05 M Tris containing 0.3% of hydrogen peroxide was applied to slides for 1 min at 20-22°C. Lymph node was used as positive control; for the negative control, the primary antibody was replaced by IgG_1_ at the same dilution as the primary antibody. Positive and negative tumour cells were counted with a pen tablet (Volito 2, Wacom Europe GmbH, Krefeld, Germany). PI was expressed as the percentage of positive tumour cells with respect to the total number of cells; counts were performed by two pathologists to ensure uniformity. A minimum of 1000 cells were counted per case.

### Statistical analysis

For statistical data evaluation, the software GraphPad PRISM 5 version 5.01 (GraphPad Software Inc, San Diego, CA, USA). was used. Continuous variables were subjected to the D’Agostino-Pearson test to determine sample distribution. Differences between the means of aglepristone-treated tumours before and after treatment were assessed by paired t-test when data were normally distributed, and otherwise by the Wilcoxon test. The agreement between RT-qPCR and IHC findings was estimated using Cohen’s κ coefficient. A P value < 0.05 was regarded as statistically significant.

## Results

### PR protein expression correlates with mRNA expression

**Figure 1:**
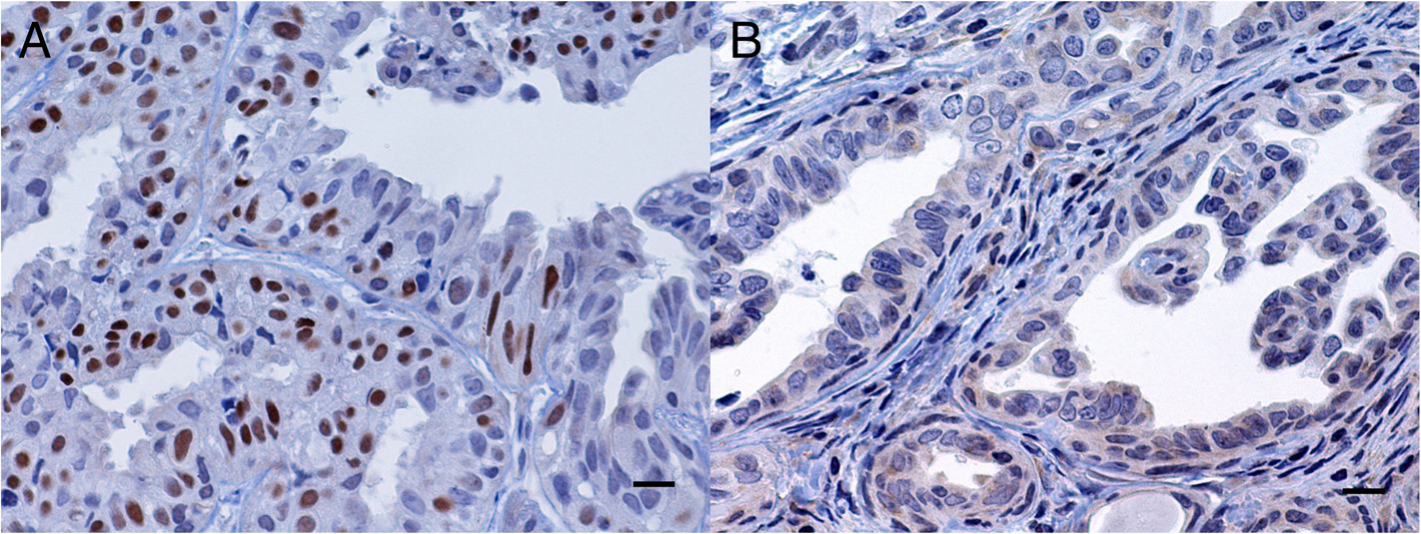
**Simple tubular mammary carcinoma.** Inmunohistochemical PR labelling is seen in the nuclei of tumour epithelial cells. A strong PR + tumour at day 1 **(A)** and a PR - tumour at day 15 **(B)** [3]. ABC immunohistochemical method. Bar = 10 μm.

**Figure 2:**
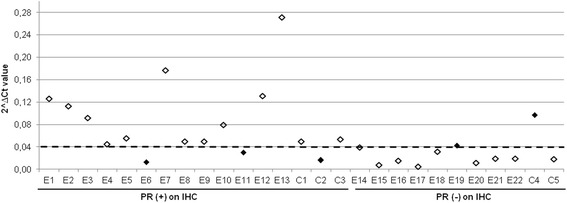
**Comparison of RT-qPCR values versus immunohistochemical assessment in the 27 samples.** The Y-axis shows PR mRNA expression values and the X-axis the PR (+) and (−) samples by IHC. The cut-off for PR (+) status by RT-qPCR is indicated by the horizontal broken line. Black diamonds show the discrepancy between the two methods.

The PR positive-status cut-off value on RT-qPCR was set at 0.04. The concordance rates at this cut-off were the highest for IHC assay (Figure 1 [3]) with a Kappa index of 0.6 (Figure 2).

### PR, PRA and PRB mRNA expression levels

Before treatment, PR mRNA values ranged from 0 to 0.27 (0.07 ± 0.01) while after treatment they lay between 0.01 and 0.15 (0.05 ± 0.01). According to RT-qPCR results, 60% of tumours in the control group (n = 3, 2 complex and 1 carcinoma in benign tumour) and 55% of tumours in the experimental group (n = 12, 8 complex, 3 carcinomas in benign tumour and 1 simple carcinoma) were classed as PR-positive prior to treatment. Complex carcinomas and carcinomas in benign tumour had similar PR mRNA (0.075 and 0.069, respectively) whereas simple carcinomas displayed the lowest levels (0.016, p = 0.002 and p = 0.04, respectively). A significant decrease in PR mRNA expression was noted after treatment in PR-positive tumours in the aglepristone-treated group alone (p = 0.001) (Table 2), whereas tumours in the control group and PR-negative tumours in the aglepristone-treated group exhibited no significant change. Overall, PR expression in PR-positive tumours in the aglepristone-treated group was reduced 2.28-fold due to treatment.

**Table 2:** **Total PR mRNA expression before and after treatment as a function of PR status on day 1**

		**Median ± SD of PR expression (2^-∆Ct)**	
	**PR status at day 1**	**Day 1**	**Day 15**
Control group	PR+	0.05 ± 0.02	0.04 ± 0.06
	PR-	0.02 ± 0.00	0.03 ± 0.00
Treated group	PR+	0.09 ± 0.07	0.03 ± 0.03*
	PR-	0.02 ± 0.01	0.03 ± 0.05

PR: Progesterone receptor; SD: Standard deviation; PR+: Progesterone receptor positive; PR-: Progesterone receptor negative. *P < 0.05.

Before treatment, PRA mRNA values ranged from 0 to 0.21 (0.046 ± 0.1) and PRB mRNA values from 0 to 0.06 (0.02 ± 0.002). The figures after treatment were 0.01 to 0.11 (0.03 ± 0.03) for PRA and 0 to 0.06 (0.01 ± 0.01) for PRB. In 48% of samples (n = 13, 8 complex carcinomas, 4 carcinomas in benign tumour and 1 simple carcinoma), PRA mRNA expression was between 3 and 10 times higher than PRB mRNA expression on day 1; PRA expression was also significantly higher than PRB expression in 80% of tumours (n = 4) in the control group and 82% in the experimental group (n = 18) (P = 0.003). After treatment, a significant decrease in PRA expression (2-fold changes with respect to pre-treatment samples) was observed in PRA-positive tumours in the aglepristone-treated group alone (p = 0.001) (Table 3). PRA-negative tumours and tumours in the control group showed no significant changes after treatment. PRB expression was not affected by aglepristone treatment in any sample (Table 4). No statistical differences were observed as a function of oestrus phase or clinical stage, or between levels of PR and PR isoform expression by RT-qPCR.

**Table 3:** **PRA mRNA expression before and after treatment as a function of PRA status on day 1**

		**Median ± SD of PRA expression (2^∆-Ct)**	
	**PRA status at day 1**	**Day 1**	**Day 15**
Control group	PRA+	0.05 ± 0.02	0.08 ± 0.06
	PRA-	0.01 ± 0.01	0.03 ± 0.00
Treated group	PRA+	0.08 ± 0.05	0.04 ± 0.02*
	PRA-	0.01 ± 0.01	0.02 ± 0.04

PRA: Progesterone receptor isoform A; SD: Standard deviation; PRA+: Progesterone receptor isoform A positive; PRA-: Progesterone receptor isoform A negative. *P < 0.05.

**Table 4:** **PRB mRNA expression before and after treatment as a function of PRB status on day 1**

		**Median ± SD of PRB expression (2^-∆Ct)**	
	**PRB status at day 1**	**Day 1**	**Day 15**
Control group	PRB+	0.014 ± 0.01	0.010 ± 0.02
	PRB-	0.007 ± 0.00	0.007 ± 0.001
Treated group	PRB+	0.014 ± 0.01	0.011 ± 0.01
	PRB-	0.006 ± 0.002	0.006 ± 0.003

PRB: Progesterone receptor isoform B; SD: Standard deviation; PRB+: Progesterone receptor isoform B positive; PRB-: Progesterone receptor isoform B negative.

### Proliferation index and PR, PRA and PRB expression levels

**Figure 3:**
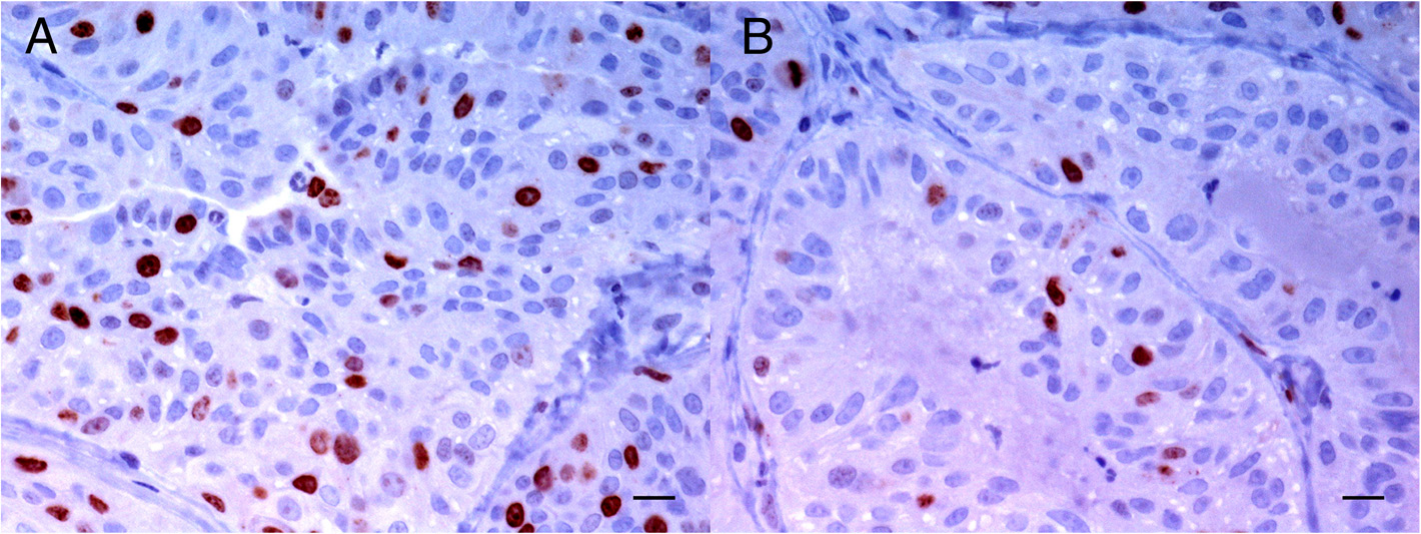
**Simple solid mammary carcinoma.** Immunohistochemical Ki67 labelling is seen in the nuclei of tumour epithelial cells at day 1 **(A)** and at day 15 **(B)**. A significant decrease of Ki67 labelling is observed at day 15 in PR-positive treated carcinomas [3]. ABC immunohistochemical method. Bar = 10 μm.

**Figure 4:**
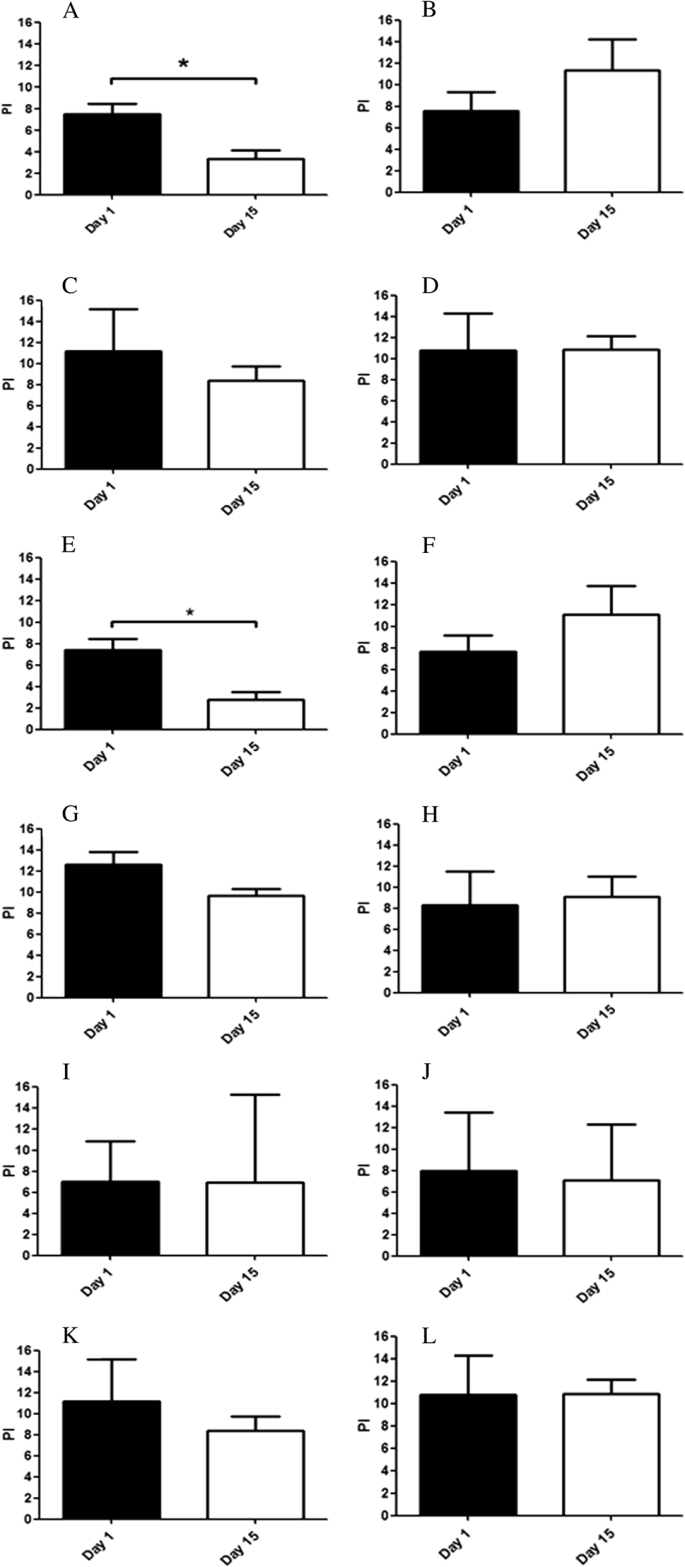
**Proliferation index.** Effect of aglepristone treatment on the percentage of Ki67-labelled cells in PR-positive treated **(A)**, PR-negative treated **(B)**, PR-positive control **(C)**, PR-negative control **(D)**, PRA-positive treated **(E)**, PRA-negative treated **(F)**, PRA-positive control **(G)**, PRA-negative control **(H)**, PRB-positive treated **(I)**, PRB-negative treated **(J)**, PRB-positive control **(K)**, PRB-negative control **(L)** canine mammary carcinomas at day 1 and day 15. *P < 0.05.

A significant decrease in PI after aglepristone treatment was observed in PR- and PRA-positive tumours in the treated group alone (P = 0.007, P = 0.01, respectively) (Figures 3 and 4). A reduction of ≥ 20% in the PI was recorded in 62% of these cases (n = 8). No significant changes in the PI were found as a function of PRB expression (Figure 4). Moreover, no statistically- significant differences in PI were observed as a function of histological tumour type or oestrus phase.

## Discussion

These findings confirm previous reports indicating a direct link between PR labelling and the antiproliferative effect of aglepristone in canine mammary carcinoma [3], and suggest that this effect might be mediated by PRA. Results for PR expression levels in canine mammary tumours are in agreement with those previously reported using IHC [3]. The epitope detected by the antibody clone used for this study (extreme C-terminus of human PR) is identical to that of canine PR, and can thus be used with dogs (http://blast.ncbi.nlm.nih.gov). Furthermore, IHC and RT-qPCR techniques demonstrated a high level of agreement for PR expression, as reported in human studies [12]. Complex carcinomas and carcinomas in benign tumour displayed higher PR expression than simple carcinomas, but no significant differences in PR expression were observed with either technique as a function of oestrus phase. Slight differences in the intensity of PR labelling by IHC have been reported in normal mammary gland (moderate to strong during oestrus and dioestrus compared with anoestrus) but the small number of animals and the lack of proestrus samples were constraints in this study [13].

The clinical impact of these findings relates to the potential use of antiprogestins in the treatment of breast cancer and to the potential prognostic value of PR isoform expression. In women, more than 70% of breast cancers express oestrogen receptors (ER) and PR, and are thus eligible for adjuvant endocrine therapy [5]. This therapy is designed to target ER either by using ER modulators or by inhibiting the endogenous synthesis of 17β-estradiol with aromatase inhibitors. However, recent research also points to PR as a therapeutic target [5,7]. In this respect, antiprogestins—either alone or in conjunction with antiestrogens—have been shown to exert an inhibitory effect in various experimental breast cancer models [7,14]. Around 75% of canine mammary carcinomas are PR positive [2] but, unlike in women, adjuvant endocrine treatment aimed at blocking the PR is not currently used. The dog has been proposed as a natural model for human breast cancer [15]. At the same time, dogs with mammary cancer may benefit from these findings, since tumours with PR and PRA expression at day 1 exhibited a significant decrease in the proliferation index after aglepristone treatment [3]. In human breast cancer, a number of studies have associated the inhibitory effect of antiprogestins with PRA but not with PRB expression [7]. A down-regulation of PRA has been reported, as in canine carcinomas, and has been cited as a possible explanation for the antiproliferative effects of antiprogestins. The absence of significant changes in PI and PRB mRNA levels in the tumours analysed here suggests that PRA mediates the antiproliferative effect of aglepristone, thus highlighting the differential roles of PRA and PRB in the canine mammary gland. These findings therefore suggest that the differential expression of PRA and PRB is critical for an appropriate therapeutic response to antiprogestins. This appears to be the first evidence of a link between PR isoform expression and proliferation in canine mammary carcinomas.

In the normal human breast, PRA and PRB are generally expressed at similar levels. However, in breast cancers, their ratio is deregulated, with a predominance of PRA over PRB [5]. In this study, most canine mammary carcinomas showed higher PRA than PRB expression regardless of histological tumour type, oestrus phase and tumour clinical stage. These results are in agreement with those reported in human breast cancer as well as in the few samples of mammary tumours studied in dogs [7,4,6]. Human studies suggest that elevated PRA expression is generally associated with a poor prognosis, and there is evidence that genetic predisposition to cancer development due to mutations in *BRCA1* or *BRCA2* genes leads to PRA overexpression, which may play a role in disease progression [16,17]. Disrupted PRA/PRB expression has been reported in endometrial cancers, and research suggests that cancers with an elevated PRA/PRB ratio are also associated with a poor prognosis [18]. However, the mechanisms via which these PR isoforms contribute to tumour genesis are not yet fully understood.

Limitations of the present study were the total number of samples used and the balance between experimental and control cases. Nevertheless, the findings provide new insights regarding PR and PR isoform expression in the context of hormone treatment. Further research with a larger number of samples is required in order to establish what triggers the mechanisms underlying the antiproliferative effect of aglepristone, and to clarify the specific role of each PR isoform.

## Conclusions

Results showed that neoadjuvant treatment of canine mammary carcinomas with the antiprogestin aglepristone reduced cell proliferation in those tumours classed as PR-positive by the RT-qPCR method. The fact that aglepristone also exerted the same effect in PRA-positive tumours and not in PRB-positive tumours, suggests that the antiproliferative effect of aglepristone in canine mammary carcinoma is probably mediated by PRA.

## Additional file

Additional file 1:
**All the supporting information is included as additional files.**

## Abbreviations

IHC: Immunohistochemical
PR: Progesterone receptor
PRA: Progesterone receptor isoform A
PRB: Progesterone receptor isoform B
FFPE: Formalin-fixed paraffin-embedded
∆∆Ct: Comparative Ct method
PI: Proliferation index
ER: Oestrogen receptor

## References

[1] Støvring M, Moe L, Glattre E (1997). A population-based case–control study of canine mammary tumours and clinical use of medroxiprogesterone acetate. APMIS.

[2] Martín de las Mulas J, Millán Y, Dios R (2005). A prospective analysis of immunohistochemically determined estrogen receptor alpha and progesterone receptor expression and host and tumour factors as predictors of disease-free period in mammary tumours of the dog. Vet Pathol.

[3] Guil-Luna S, Sánchez-Céspedes R, Millán Y, De Andrés FJ, Domingo V, Guscetti F, Martín de las Mulas J (2011). Aglepristone decreases proliferation in progesterone receptor-positive canine mammary carcinomas. J Vet Intern Med.

[4] Latingan-van Leeuwen IS, van Garderen E, Rutteman GR, Mol JA (2000). Cloning and cellular localization of the canine progesterone receptor: co-localization with growth hormone in the mammary gland. J Steroid Biochem Mol Biol.

[5] Lanari C, Wargon V, Rojas P, Molinolo AA (2012). Antiprogestins in breast cancer treatment: are we ready?. Endocr Relat Cancer.

[6] Gracanin A, Gier J, Zegers K, Bominaar M, Rutteman GR, Schaesfers-Okkens AC, Kooistra HS, Mol JA (2012). Progesterone receptor isoforms in the mammary gland of cats and dogs. Reprod Domest Anim.

[7] Wargon V, Helguero LA, Bolado J, Rojas P, Novaro V, Molinolo A, Lanari C (2009). Reversal of antiprogestin resistance and progesterone receptor isoform ratio in acquired resistant mammary carcinomas. Breast Cancer Res Treat.

[8] Guil-Luna S, Stenvang J, Brünner N, Sánchez-Céspedes R, Millán Y, Gómez-Laguna J, Mulas JM (2013). Progesterone receptor isoforms analysis by RTq-PCR in formalin-fixed paraffin-embedded canine mammary dysplasias and tumours. Vet Pathol.

[9] Misdorp W, Else RW, Hellmén E, Lipscomb TP (1999). Histological classification of mammary tumors of the dog and the cat. World Health Organization International Histological Classification of Tumors of Domestic Animals. Volume 7. 2nd series.

[10] Schmittgen TD, Livak KJ (2008). Analyzing real-time PCR data by the comparative Ct method. Nat Protoc.

[11] Hayashi A, Tanabe A, Kawabe S, Hayashi M, Yuquchi H, Yamashita Y, Okuda K, Ohmichi M (2012). Dienogest increases the progesterone receptor isoform B/A ratio in patients with ovarian endometriosis. J Ovarian Res.

[12] Oda M, Arihiro K, Kataoka T, Osaki A, Asahara T, Ohdan H (2010). Comparison of immunohistochemistry assays and real-time reverse transcription-polymerase chain reaction for analyzing hormone receptor status in human breast carcinoma. Pathol Int.

[13] Chandra SA, Cline MJ, Adler RR (2010). Cyclic morphological changes in the beagle mammary gland. Toxicol Pathol.

[14] Gaddy VT, Barrett JT, Delk JN, Kallab AM, Porter AG, Schoenlein PV (2004). Mifepristone induces growth arrest, caspase activation, and apoptosis of estrogen receptor expression, antiestrogen-resistant breast cancer cells. Clin Cancer Res.

[15] Pinho SS, Carvalho S, Cabral J, Reis CA, Gärtner F (2012). Canine tumors: a spontaneous animal model of human carcinogenesis. Transl Res.

[16] Mote PA, Leary JA, Avery KA, Sandelin K, Chenevix-Trench G, Kirk JA, Clarke CL, KConFab Investigators (2004). Germ-line mutations in BRCA1 and BRCA2 in the normal breast are associated with altered expression of estrogen-responsive proteins and the predominance of progesterone receptor A. Genes Chromosomes Cancer.

[17] Poole AJ, Li Y, Kim Y, Lin SC, Lee WH (2006). Prevention of BRCA1-mediated mammary tumourigenesis in mice by a progesterone antagonist. Science.

[18] Arnett-Mansfield RL, DeFazio A, Wain GV, Jaworski RC, Byth K (2001). Relative expression of progesterone receptors A and B in endometrioid cancers of the endometrium. Cancer Res.

