# Supplementary material for: Progesterone receptor isoform A may regulate the effects of neoadjuvant aglepristone in canine mammary carcinoma
Source: BMC Vet Res. 2014 Dec 17;10:296. doi: 10.1186/s12917-014-0296-2 (PMC4280049; doi:10.1186/s12917-014-0296-2)
Supplement: Additional file 1: — All the supporting information is included as additional files. [file 12917_2014_296_MOESM1_ESM.docx]

***Quantitative Real Time PCR assays (qPCR)***

*Primer design*

The canine PR genomic sequence was obtained from NCBI GenBank database under the gene ID NM_001003074 (http://www.ncbi.nlm.nih.gov/). Primer pairs were designed using Primer3Plus (http://primer3plus.com/cgi-bin/dev/primer3plus.cgi) to target the coding regions of both canine isoforms which were previously described by Lantinga-van Leeuwen et al. (2000). In addition, the hypoxanthine phosphoribosyl-trasnferase 1 (HPRT1: NM_001003357.1) and canine ribosomal protein L32 (RPL32: NM_001252169.1) were selected as canine housekeeping genes and primer pairs were designed with the same tool. These two genes have shown to be suitable reference genes as internal controls for RT-qPCR in canine mammary tumors.^1,2^


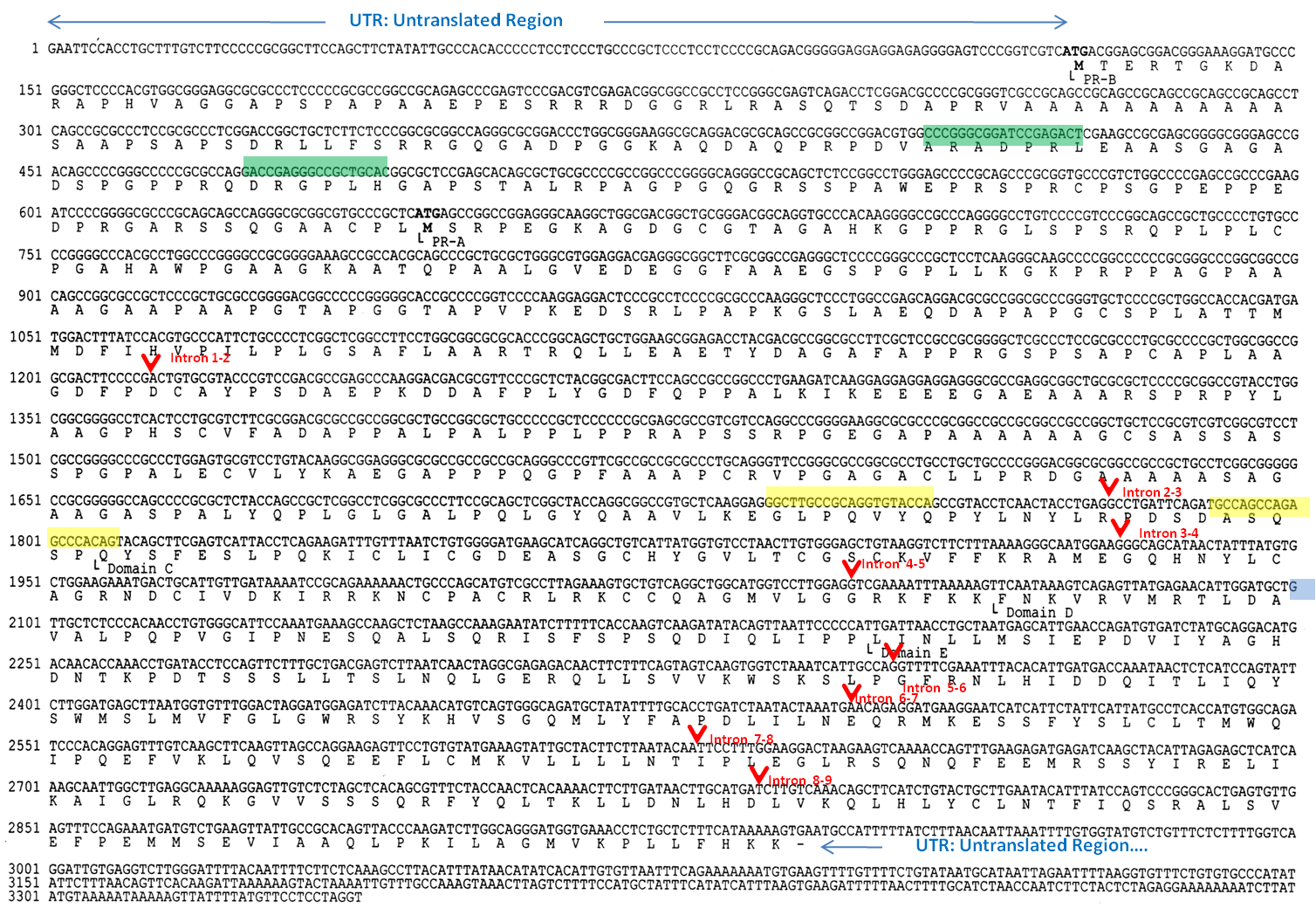


**Figure 1.** Complete canine PR sequence (figure modified from Latingan-van Leeuwen et al., 2000). In green color, target sequence for isoform B and in yellow color, target sequence for total PR.

***References***

1. Johnson CM, Yang S, Sellins KS et al. **Selection of HPRT primers as controls for determination of mRNA expression in dogs by RT-PCR**. *Veterinary Immunology and Immunopathology.* 2004, **99**:47-51.
2. 7. Etschmann B, Wilcken B, Stoevesand K et al. **Selection of reference genes for quantitative real-time PCR analysis in canine mammary tumors using the GeNorm Algorithm.** *Veterinary Pathology*. 2006, **43**: 934-942.
